# Differentiation Potential of Mesenchymal Stem/Stromal Cells Is Altered by Intrauterine Growth Restriction

**DOI:** 10.3389/fvets.2020.558905

**Published:** 2020-11-05

**Authors:** Emma L. Weatherall, Viktorija Avilkina, Yennifer Cortes-Araya, Susan Dan-Jumbo, Claire Stenhouse, Francesc X. Donadeu, Cristina L. Esteves

**Affiliations:** ^1^The Roslin Institute and The Royal (DICK) School of Veterinary Studies (R(D)SVS), The University of Edinburgh, Edinburgh, United Kingdom; ^2^The Euan Macdonald Centre, The University of Edinburgh, Edinburgh, United Kingdom

**Keywords:** MSC - 51 (SCM), adipogenesis, osteogenesis, chondrogenesis, pig, IUGR, regenerative

## Abstract

Consistency in clinical outcomes is key to the success of therapeutic Mesenchymal Stem/Stromal cells (MSCs) in regenerative medicine. MSCs are used to treat both humans and companion animals (horses, dogs, and cats). The properties of MSC preparations can vary significantly with factors including tissue of origin, donor age or health status. We studied the effects of developmental programming associated with intrauterine growth restriction (IUGR) on MSC properties, particularly related to multipotency. IUGR results from inadequate uterine capacity and placental insufficiency of multifactorial origin. Both companion animals (horses, dogs, cats) and livestock (pigs, sheep, cattle) can be affected by IUGR resulting in decreased body size and other associated changes that can include, alterations in musculoskeletal development and composition, and increased adiposity. Therefore, we hypothesized that this dysregulation occurs at the level of MSCs, with the cells from IUGR animals being more prone to differentiate into adipocytes and less to other lineages such as chondrocytes and osteocytes compared to those obtained from normal animals. IUGR has consequences on health and performance in adult life and in the case of farm animals, on meat quality. In humans, IUGR is linked to increased risk of metabolic (type 2 diabetes) and other diseases (cardiovascular), later in life. Here, we studied porcine MSCs where IUGR occurs spontaneously, and shows features that recapitulate human IUGR. We compared the properties of adipose-derived MSCs from IUGR (IUGR-MSCs) and Normal (Normal-MSCs) new-born pig littermates. Both MSC types grew clonally and expressed typical MSC markers (CD105, CD90, CD44) at similar levels. Importantly, tri-lineage differentiation capacity was significantly altered by IUGR. IUGR-MSCs had higher adipogenic capacity than Normal-MSCs as evidenced by higher adipocyte content and expression of the adipogenic transcripts, PPARγ and FABP4 (*P* < 0.05). A similar trend was observed for fibrogenesis, where, upon differentiation, IUGR-MSCs expressed significantly higher levels of COL1A1 (*P* < 0.03) than Normal-MSCs. In contrast, chondrogenic and osteogenic potential were decreased in IUGR-MSCs as shown by a smaller chondrocyte pellet and osteocyte staining, and lower expression of SOX9 (*P* < 0.05) and RUNX2 (*P* < 0.02), respectively. In conclusion, the regenerative potential of MSCs appears to be determined prenatally in IUGR and this should be taken into account when selecting cell donors in regenerative therapy programmes both in humans and companion animals.

## Introduction

Mesenchymal Stem/Stromal cells (MSCs) are used in regenerative therapies both in humans (1) and in veterinary species (horses, dogs, and cats) (2–6). MSCs are typically obtained from bone marrow and adipose tissue, but other sources such as umbilical cord are also becoming more regularly investigated (7, 8). As defined by the International Society for Cellular Therapy (ISCT) (9), MSCs grow adherent to plastic and clonally, and express a group of surface markers, CD105, CD73, and CD90, and lack expression of CD45, CD34, CD14 or CD11b, CD79α or CD19 and HLA-DR. In addition, MSCs are multipotent having the capacity of differentiating into cells of the mesenchymal lineage (adipocytes, osteocytes, and chondrocytes), and these features have been explored for regenerative therapeutic purposes. These cells produce trophic factors which are relevant to the repair processes *in vivo*, and therefore are considered as “Medicinal Signaling Cells” (10).

Different factors, such as donor (11) and age (12), can have considerable impact on MSC properties and clinical outcome, increasing the difficulty of MSC standardization, commercialization and therapeutic use. In addition, donor disease, such as diabetes and osteoarthritis (13, 14) can have a detrimental impact on the quality of MSC preparations from the stromal vascular fraction, which are heterogeneous cells obtained from extracts containing stem and other cells, such as those of endothelial and hematopoietic origin.

In IUGR, which is observed both in humans and veterinary species, the fetus fails to reach its full genetic growth potential (15) resulting in an increased risk of premature offspring death. This is the case of “runt” puppies, which may actually be preferred by some owners due to their appearance, but are at increased risk of presenting adverse developmental and metabolic features in adulthood (16, 17). Likewise, IUGR can affect equine health and performance in adulthood by impacting negatively on muscle and skeleton development and function, metabolism and pulmonary efficiency (18, 19).

In humans, IUGR occurs in about 10–40% pregnancies (20–23) resulting in delayed fetal growth and small weight at birth often associated with placental insufficiency as a result of maternal advanced diabetes, anemia and high blood pressure, malnutrition, multiple gestation of two or more fetuses, infection, alcohol consumption and use of recreation drugs (24, 25). Epidemiological data have shown a clear link between IUGR and obesity, and an increased risk of type 2 diabetes and cardiovascular disease in adulthood (26–28).

IUGR has been reported to affect cell growth and adipogenesis differentiation properties of stem cells (29, 30), which may underlie altered developmental phenotype and predisposition to later disease in IUGR individuals. However, the impact of IUGR on MSC properties is still unclear.

Pigs offer a relevant model to study IUGR. In pigs, IUGR occurs spontaneously, and recapitulates human IUGR features, including altered body development characterized by reduced musculoskeletal growth and a tendency to accumulate body fat post-nataly (31–33), as it is also observed in other models (16, 34). Using the IUGR pig model (16, 35, 36), and in order to assess the effect of developmental programming on MSCs, we compared the properties of MSCs obtained from IUGR and normal newborn pig littermates. Considering the IUGR phenotype, we hypothesized that IUGR impacts on MSCs properties by increasing adipogenesis and fibrogenesis and decreasing the ability of cell differentiation into other lineages such as osteogenesis and chondrogenesis.

## Materials and Methods

### Samples

Subcutaneous adipose tissue samples were obtained immediately postmortem from three pairs of littermates Large White × Landrace, 1–7 days old, in accordance to the UK Home Office Animals (Scientific Procedures) Act 1986, schedule 1, by decapitation following administration of isoflurane and euthatal.

Normal and IUGR (birthweight below two standard deviations the average litter weight) mates were collected from each litter. Tissue samples were kept on ice in PBS with amphotericin B and 1% Penicillin and Streptomycin (P/S; Life Technologies-Thermo Fisher Scientific) until cell extraction <1 h after collection.

### Cell Extraction and Culture

Adipose tissue was minced and digested in the presence of collagenase II (1 mg/ml; Thermo Fisher Scientific), Bovine Serum Albumin (3.5%) and DNase (40 μg/ml; Sigma-Aldrich) for 1 h at 37°C with gentle rotation at 100 rpm (37, 38). Collagenase activity was stopped with Dulbecco's Modified Eagle Medium (DMEM; D5796; Sigma), 20% FBS (Thermo Fisher Scientific). After removing the lipid layer, the stromal vascular fraction was filtered through a 40 μm cut-off sieve. Cells were cultured initially in DMEM High Glucose supplemented with 20% FBS (Life Technologies-Thermo Fisher Scientific) and 50 μg/ml basic fibroblast growth factor (bFGF; ReproTech), and then, in the subsequent passages, bFGF was removed from the medium and FBS reduced to 10%.

### Cell Growth Analyses

Doubling time was calculated using the formula:

$$Doubling\;Time=\frac{\text{Time in culture }\times \text{ log}(2)}{\text{log}(\text{Final number})-\text{log}(\text{Initial number})}$$

Where “Initial” and “Final” numbers refer to number of cells at seeding and harvesting, respectively. To test the ability of cells to grow clonally, 250, 500, and 1,000 cells from each population at passage 3 were plated in 6-well plates, and cultured for 10 days. Cells were then fixed with paraformaldehyde (2%; 30 min) and washed with PBS P/S before staining with 1% Crystal violet in 100% Methanol.

### Analyses of Cell Differentiation

#### Adipogenesis

To induce adipogenesis cells were seeded on collagen (0.2%) in 24-well plates (50,000 cells/well) and grown until confluence as previously described (5). Cells were kept for 5 days in differentiation medium consisting of DMEM supplemented with 7% Rabbit Serum (Gibco), 3% FBS, 1% P/S, 1 μM dexamethasone (Sigma-Aldrich), 10 μg/ml insulin (Sigma-Aldrich) and 0.5 mM 3-isobutyl-1-methylxanthine. This was followed by 7 days of cell culture in DMEM with 10% FBS, 1% P/S and 10 μg/ml insulin. Medium was changed every 2–3 days. In order to visualize lipid accumulation, differentiated adipocytes were stained with Oil red O (0.375% prepared in isopropanol) for 10 min and imaged in a Zeiss Axiovert 25 Inverted Phase microscope, and differences between the two types of cells were assessed visually.

#### Fibrogenesis

Fibrogenesis was induced when cells reached 80% confluence by using a medium consisting of DMEM High Glucose, supplemented with 0.5% FBS, 0.1 mM Ascorbic acid, 1% P/S, 0.1 mg/ml Dextran Sulfate Sodium Salt, and 2.5 ng/ml Transforming Growth Factor β (Sigma) for 3 and 6 days, as previously described (39). However, by day 6 of differentiation cells were detaching from the cell culture vessel and dying, and these samples were not analyzed. Collagen was detected by staining cell cultures with Picrosirus Red [0.1%; (40)] and then washed thoroughly with acidified water (250 μl glacial acetic acid in 50 mL MilliQ water) and micrographs were taken in an Zeiss Axiovert 25 Inverted Phase microscope using Zen 2 software (Advanced Micro Devices), and differences between the two types of cells were assessed visually.

#### Osteogenesis

Osteogenic differentiation was induced when cells reached 90% confluence with DMEM high glucose and DMEM low glucose (50:50 v/v; Sigma-Aldrich), supplemented with 10% FBS, 100 nM dexamethasone (Sigma-Aldrich), 10 mM sodium β-glycerophosphate (Sigma-Aldrich) and 0.1 mM stabilized ascorbic acid (Sigma-Aldrich) (37). After 3 days, cells were switched to DMEM low glucose supplemented with 10% FBS, 100 nM dexamethasone, 10 mM sodium β-glycerophosphate and 0.1 mM stabilized ascorbic acid. Cells were cultured for 12 days and medium was changed every 3 days. At the end of the differentiation period cells were stained with Alizarin Red (2%; pH 4.2) for 2 h and imaged in a Zeiss Axiovert 25 Inverted Phase microscope using Zen 2 software (Advanced Micro Devices) and differences between the two types of cells were assessed visually.

#### Chondrogenesis

Chondrogenesis was induced by using the StemPro Chondrogenesis Differentiation Kit (A1007101, Thermofisher). Briefly, cells were seeded in micromasses (80,000 cells/each) and incubated for 2 h in a humidified chamber in the incubator before differentiation medium was added. After 28 days, the chondrogenic micromasses were either harvested into Trizol for gene expression measurements or fixed in paraformaldehyde (4%) for 45 min and directly stained overnight with Alcian Blue (1%; Sigma). Pellets were imaged in a Zeiss Axiovert 25 Inverted Phase microscope and radius measured with Zen 2 to calculate the spherical area (A = 4πr^2^, where A is the area and r the radius).

### Gene Expression Analysis

Gene expression analysis was performed in cells before and after differentiation. RNA was extracted using TRIzol reagent (Invitrogen), and 500 ng were reversed transcribed with Superscript III (Invitrogen). qPCR was performed using the SensiFAST SYBR Lo-ROX (BIO-94020; Bioline) in a Stratagene thermocycler with primers listed in Table 1. Relative transcript abundance was obtained using MX3005P software by extrapolating Ct values from a standard curve prepared from a sample pool, and TOP2B and RPL4 genes were used as housekeeping gene controls. Differentiation time-point data were normalized to values from undifferentiated cells (Day 0).

**Table 1:** List of primers used in this study.

**Gene**	**Forward primer**	**Reverse primer**
CD44^*^	CAGGTACGGATTCAAATATCA	ACTGGGGTGTTTGTCTCTT
	TCTCAGC	TCATCTTC
CD90^*^	GACTGCCGCCATGAGAATAC	GGTAGTGAAGCCTGATAAGTAGAG
CD105^*^	ATACAAAGGGCTCCATCATC	TGAGTGTGAGACTTCCATTC
PPARγ	CTGACCAAAGCAAAGGCGAG	GACACCCCTGAAAGATGCGA
FABP4	ACGGCTTCTTTCTCACCTTGA	AGCCCACTCCCACTTCTTTC
COL1A1	TTCTAAGCCGCGTCTCTTCC	TCTCCCTTGGGTCCCTATCG
ALP	ATGAGCTCAACCGGAACAA	GTGCCCATGGTCAATCCT
RUNX2	CAAAGCCAGAGCGGAC	AATTTGGATTTAATAGCGTGC
SOX9	TCAACCCCGACTGCGACGAG	TGGAGCAGCTGGGATGATGG
RPL4	AATTTGGATTTAATAGCGTGC	GAACTCTACGAATCTTC
TOP2B	AACTGGATGATGCTAATGCT	TGGAAAAACTCCGTCTGTCTC

* *Primers designed in a previous study (41)*.

### Statistical Analysis

Results were analyzed by Student's *t*-test or Two-way ANOVA followed by Tukey's *post hoc* test as appropriate, by using GraphPad Prism 8.2.1. Experiments were performed in triplicate and statistical significance was defined as *P* < 0.05.

## Results

### IUGR- and Normal-Cells Displayed Typical MSC Features

**Figure 1:**
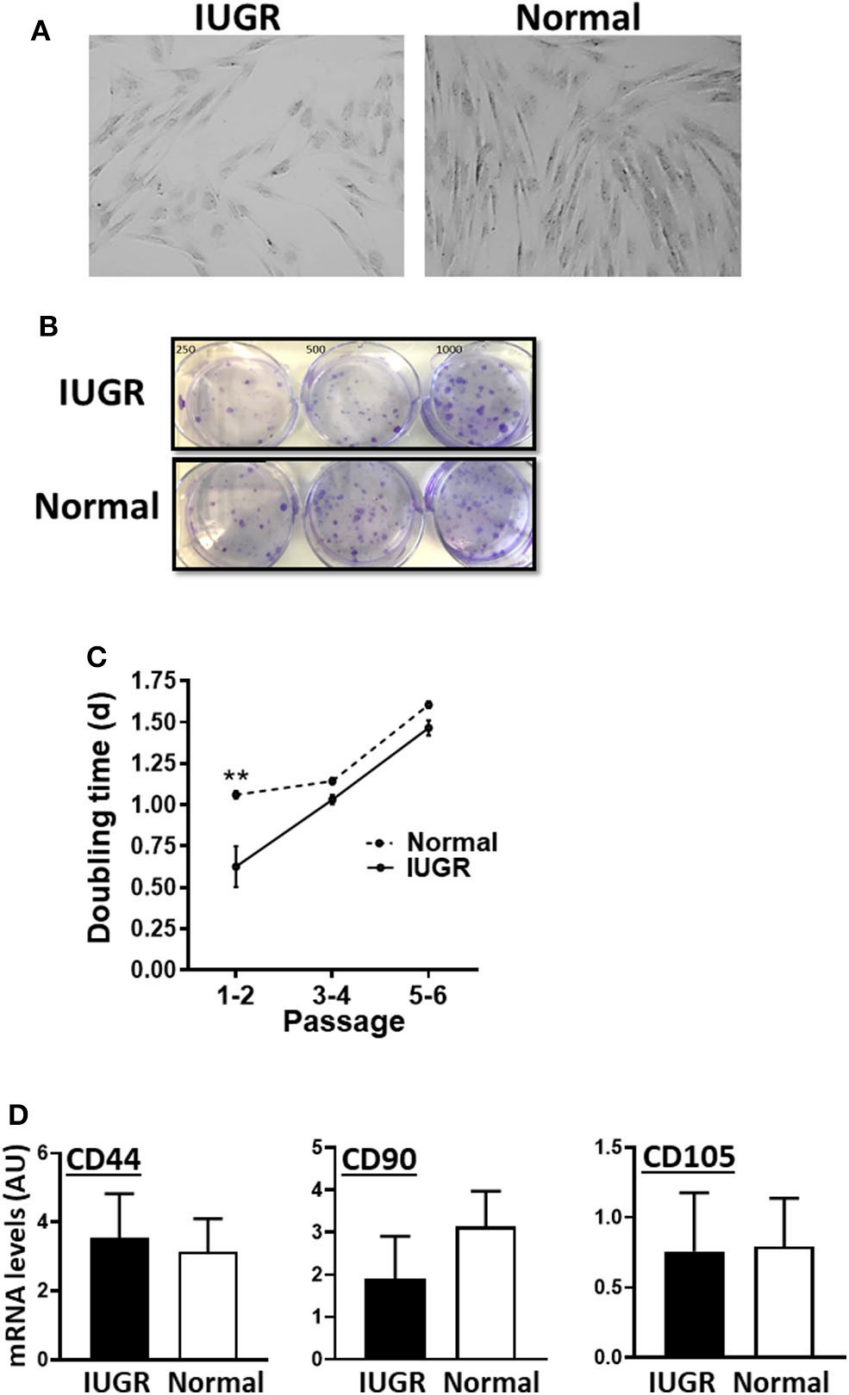
Appearance and growth of IUGR- and Normal-MSCs in culture. **(A)** Micrographs, taken at 10×, of IUGR- and Normal-MSCs displaying the typical spindle-like morphology. **(B)** Cell colonies obtained from IUGR- and Normal-MSCs after seeding 250, 500, 1,000 cells/well (left, center, and right wells) and stained with crystal violet. **(C)** Rate of cell growth expressed as doubling time for IUGR- and Normal-MSCs (dashed and continuous line, respectively) at passages 1–2 to 5–6. **indicates a significant difference (*P* = 0.001) between IUGR- and Normal-MSCs. **(D)** mRNA levels, measured by qPCR, of the MSC markers, CD44, CD90, and CD105 for IUGR- and Normal-MSCs. All results are shown as mean ± SEM; AU, arbitrary units.

MSCs from both IUGR and Normal animals presented similar morphology in culture and exhibited clonal growth (Figures 1A,B). In addition, IUGR-MSCs grew faster than Normal-MSCs but only during the first 2 passages (*P* = 0.001; Figure 1C) after which both cell types showed the same rate of division. In addition, both IUGR- and Normal-MSCs expressed the classical MSC markers, CD44, CD90, and CD105 which levels were not statistically different (Figure 1D and Supplementary Table 1; *P* = 0.8, 0.4, 0.9, respectively), while the hematopoietic cell marker CD45 was not detected in either cell type.

### IUGR-MSCs Showed Increased Adipogenesis and Fibrogenesis Compared to Normal-MSCs

**Figure 2:**
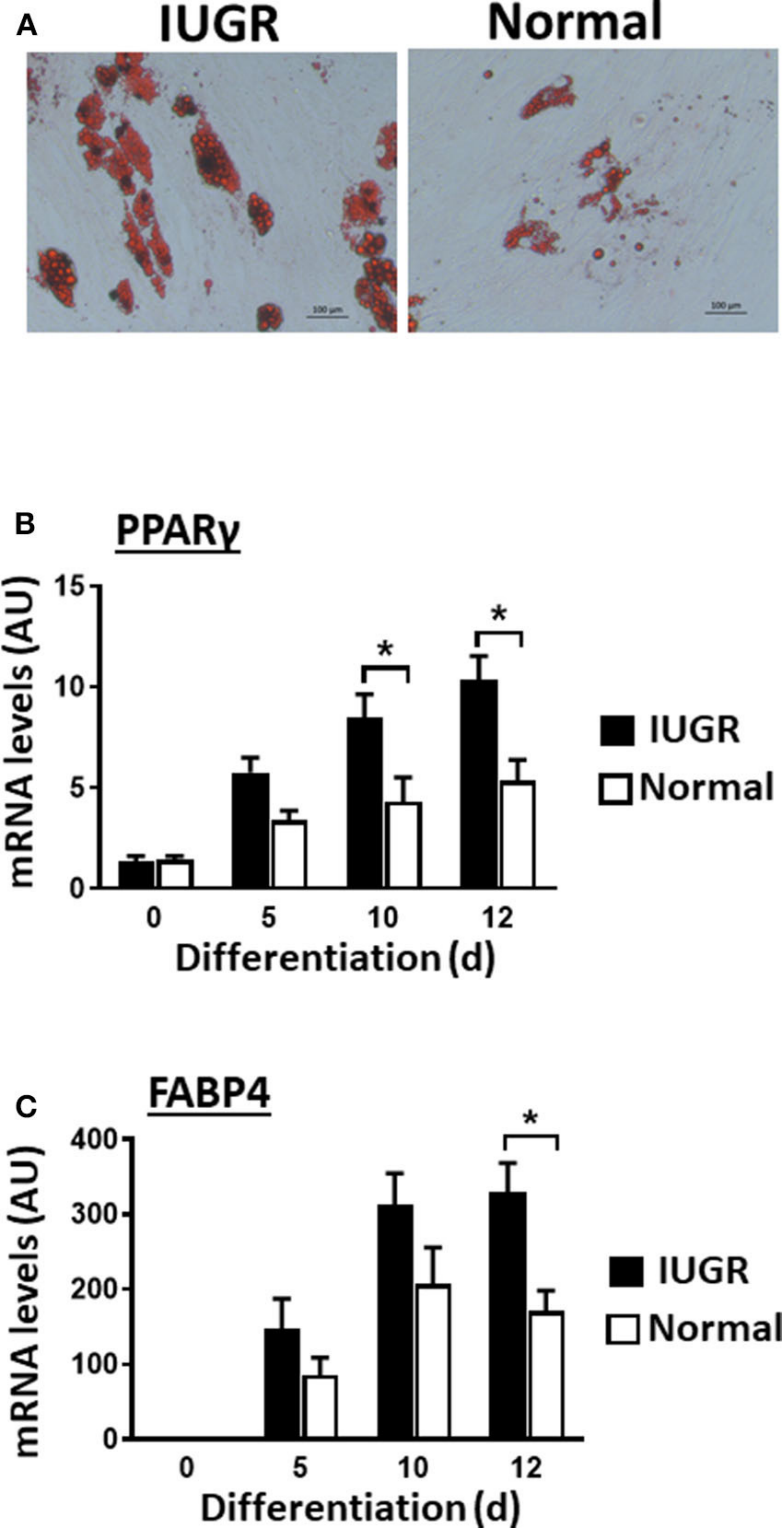
Adipogenic differentiation of IUGR- and Normal-MSCs. **(A)** Micrographs showing differentiated adipocytes of IUGR- and Normal-MSCs stained with Oil red O. Scale bars correspond to 100 μm of micrographs taken at 20×. mRNA levels, measured by qPCR, of **(B)** PPARγ and **(C)** FABP4 during adipogenic differentiation of IUGR- and Normal-MSCs. All results are shown as mean ± SEM; AU, arbitrary units. *P < 0.05, indicates differences between IUGR- and Normal-MSCs.

**Figure 3:**
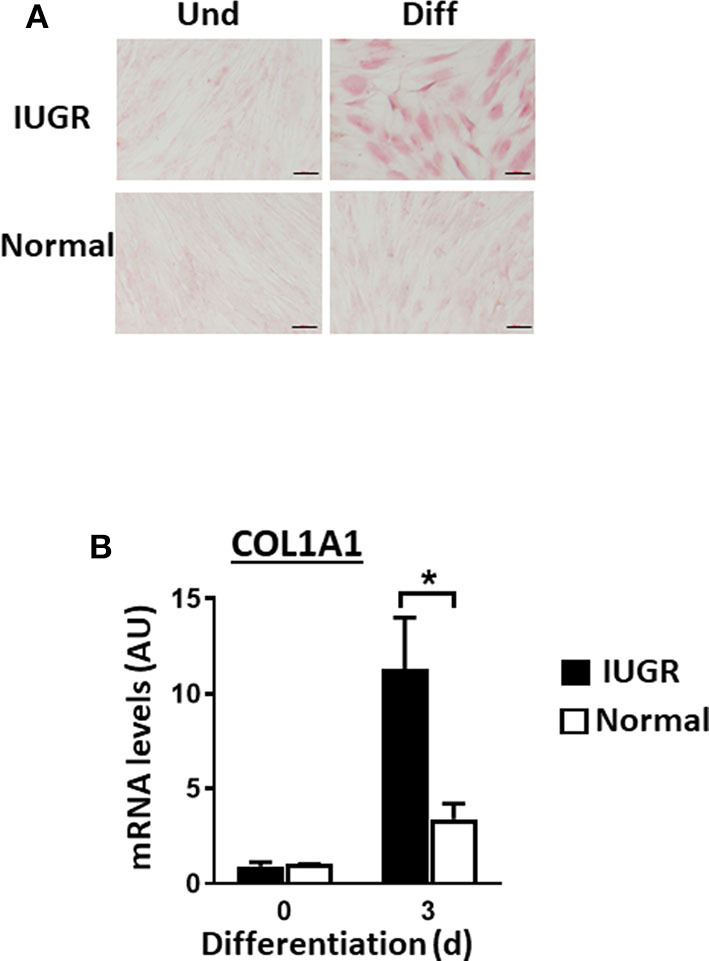
Fibrogenesis of IUGR- and Normal-MSCs. **(A)** Micrographs presenting undifferentiated (Und) and differentiated (Diff) cells from IUGR- and Normal-MSCs, stained with Picrosirius Red collagen staining. Scale bars correspond to 100 μm of micrographs taken at 20×. **(B)** mRNA levels of COL1A1, measured by qPCR, in IUGR- and Normal-MSCs before (day 0) and after 3 days of induction of fibrogenesis. All results are shown as mean ± SEM; AU, arbitrary units. *P < 0.05, indicates differences between IUGR- and Normal-MSCs.

Upon induction of adipogenesis, both MSC types produced mature adipocytes, as shown by staining of intracellular lipids with Oil Red O (Figure 2A). However, differentiation was more efficient for IUGR- than Normal-MSCs as evidenced by the expression of adipogenesis-associated genes, PPARγ and FABP4, a transcription factor and a fatty acid carrier protein, respectively (*P* < 0.05; Figures 2B,C). Likewise, fibrogenesis as evidenced by the Picrosirius Red collagen staining (Figure 3A), was more pronounced in IUGR-MSCs, as shown by higher expression of COL1A1, a major component of type I collagen (*P* < 0.03; Figure 3B), 3 days after the induction of differentiation of IUGR- compared to Normal-MSCs.

### IUGR-MSCs Showed Attenuated Osteogenesis Compared to Normal-MSCs

**Figure 4:**
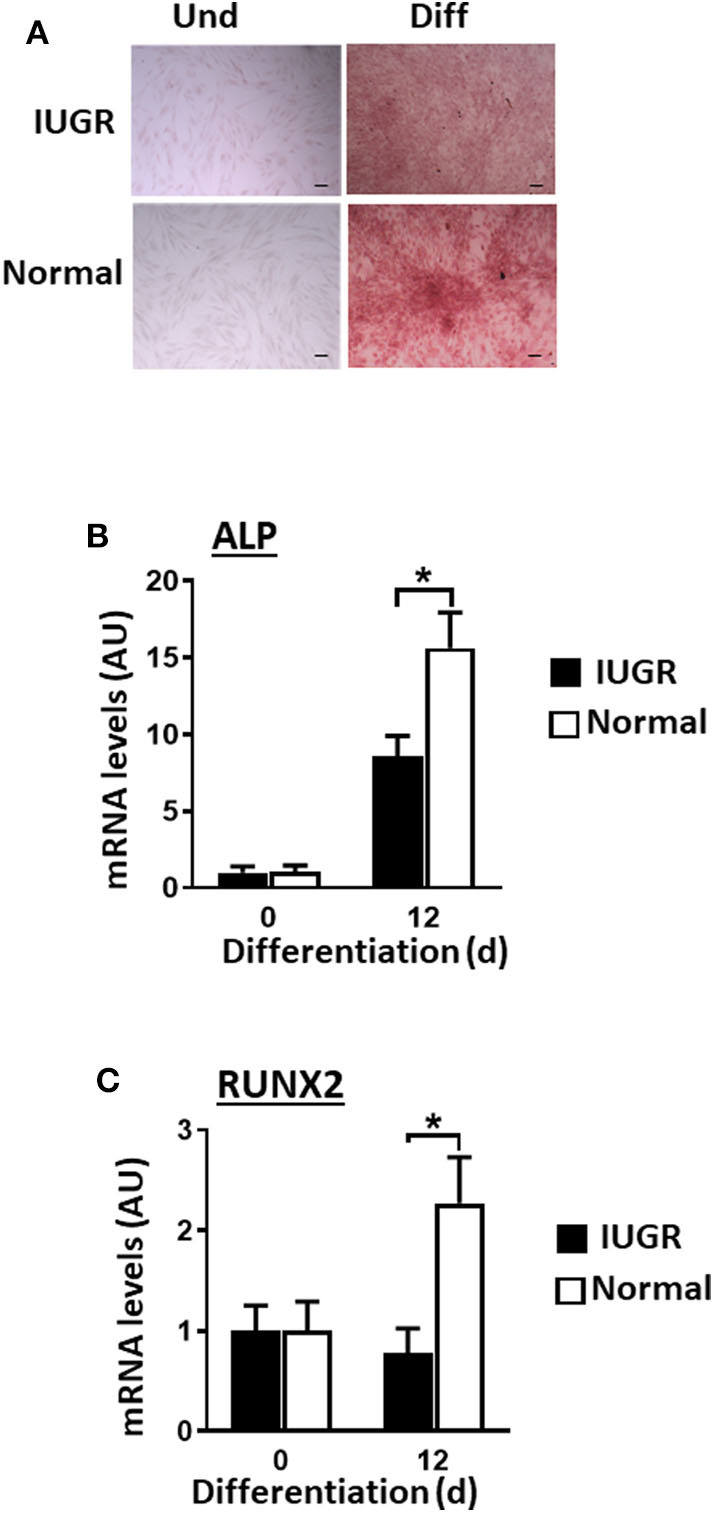
Osteogenic differentiation of IUGR- and Normal-MSCs. **(A)** Micrographs of IUGR- and Normal-MSCs stained with Alizarin Red before (Und) and after differentiation (Diff). Scale bars correspond to 100 μm of micrographs taken at 10×. mRNA levels, measured by qPCR, for **(B)** ALP and **(C)** RUNX2, before and after induction of differentiation of IUGR- and Normal-MSCs. All results are shown as mean ± SEM; AU, arbitrary units. *P < 0.05 shows differences between IUGR- and Normal-MSCs.

Contrary to what was observed for adipogenesis and fibrogenesis, IUGR-MSCs had reduced osteogenic capacity compared to Normal-MSCs, as indicated by lower expression of RUNX2 and ALP (a transcription factor and a osteogenic marker, respectively; *P* < 0.02; Figures 4B,C) after 12 days of differentiation in IUGR-MSCs. Differentiated cells stained positively with Alizarin Red (Figure 4A) and both RUNX2 and ALP genes increased during differentiation of Normal-MSCs (*P* < 0.04), however, ALP (*P* = 0.02) but not RUNX2 (*P* = 0.96) increased during differentiation of IUGR-MSCs.

### IUGR-MSCs Presented Decreased Chondrogenesis Compared to Normal-MSCs

**Figure 5:**
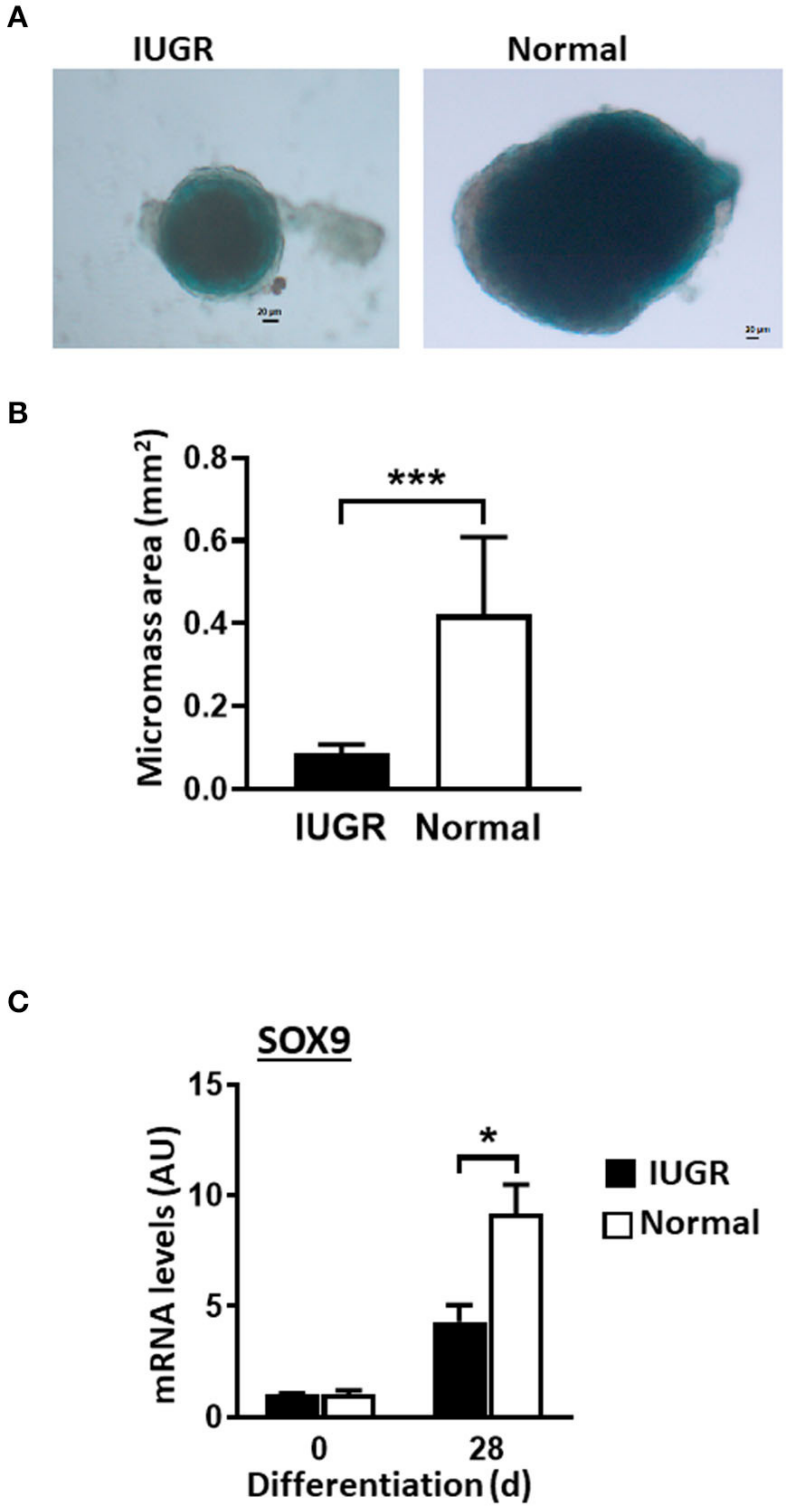
Chondrogenesis of IUGR- and Normal-MSCs. **(A)** Micrographs showing chondrogenic micromasses from IUGR- and Normal-MSC 28 days after induction of differentiation (scale bar = 20 μm, 5× magnification), stained with Alcian Blue. **(B)** Graph showing results of micromass quantification. **(C)** mRNA levels of SOX9, measured by qPCR, in IUGR- and Normal- before (0 days) and after 28 days of chondrogenic induction. All results are shown as mean ± SEM; AU, arbitrary units. *P < 0.05; ***P < 0.0001, shows differences between IUGR- and Normal-MSCs.

Both Normal- and IUGR-MSCs differentiated into chrondrocytic micromasses as evidenced by Alcian Blue staining (Figure 5A) and increased transcription factor SOX9 expression levels (*P* < 0.05). Similarly to osteogenic differentiation, chondrogenesis was more extensive in Normal- than in IUGR-MSCs as demonstrated by the larger micromasses (*P* = 0.001; Figures 5A,B) and higher expression levels of the transcription factor SOX9 (*P* < 0.05; Figure 5C) measured after 28 days of differentiation.

## Discussion

Dysregulated tissue development and a predisposition for later life disease in IUGR are thought to result from programming effects during fetal development. The implications of fetal programming on the properties of body stem cell populations and their potential for regenerative medicine are still unclear. The pig, a multiparous species, provides a well-established, pre-clinical model of cell-based tissue regeneration and engineering, gene therapy and xenotransplantation, most notably in relation to cardiovascular repair (42–45). In addition, contrary to other models such as the rat and the sheep, IUGR occurs spontaneously and with relative high frequency in pigs, with most litters containing at least one piglet that is distinctly smaller than the other littermates (35, 36, 46). Thus, comparisons between IUGR and normal phenotypes can be performed within the same genetic background in the pig (16). With this rationale, we sought to investigate the effects of fetal reprogramming on MSC properties in IUGR pigs. Our results showed that, although MSCs from IUGR share similar features with MSCs from Normal littermates, multipotency is significantly dysregulated in IUGR MSCs, with their adipogenic and fibrogenic capacity being increased in detriment of chondrogenesis and osteogenenesis, as assessed by gene expression of appropriate markers and chemical stainings of differentiated cells (which were only visually evaluated). This indicates that stem cell programming during fetal development impacts negatively on the quality of MSCs by compromising their differentiation potential. These are important aspects that should be taken into consideration during selection of MSCs for regenerative therapy applications.

Porcine MSCs have been characterized (47, 48), and they display features, in terms of marker expression and multipotency, that are similar to those of companion animals and human MSCs (49). Here, we showed that, similar to Normal-MSCs, IUGR-MSCs displayed classical features of human and porcine MSCs (49–51) namely of CD marker expression, as defined by ISCT (9), and, except for a faster initial cell growth of Normal-MSCs, there were no differences between the two cell types. Others studies, using different IUGR models showed variable results regarding MSC growth; in the rat, food-restriction caused increased cell proliferation of bone marrow MSCs (29), whilst in the sheep, bone marrow MSC proliferation was reportedly reduced with poor maternal nutrition (30).

IUGR in humans (21, 28) and animals, namely pig (33, 52, 53), sheep (31) and rat (54), results in increased body adiposity and fibrosis (55, 56). In contrast, IUGR has a negative impact on bone (34, 57), cartilage (58) and skeletal muscle development (33, 59) as observed in horses (18, 19) and dogs (17, 60). Our results indicated an increased predisposition of IUGR-MSCs for adipogenesis and fibrogenesis which suggested that IUGR programmes the adult stem cell niche during fetal development, and this may underpin the increased accumulation of fat and fibrous tissue reported in IUGR individuals, later in life (61). This is likely to be a generalized body effect, i.e., not limited to adipose-derived stem cells as the same effect was observed by us in cells obtained from skeletal muscle (unpublished results) and in bone marrow IUGR-MSCs in other studies (29). In addition, we showed that IUGR MSCs had decreased osteogenic and chondrogenic capacities, in agreement with observations in different IUGR models *in vivo* (16). IUGR may also impact other MSC properties which were not part of the objectives of the present work, such as those related to angiogenesis and immunomodulation. A negative association between adipogenesis and osteogenesis in the body is also observed in other settings, such as aging, osteoporosis and obesity (62, 63). It is conceivable that in some instances these effects may be linked to IUGR, or at least, they are likely to be exacerbated in IUGR subjects with consequences for health, tissue repair and healing and, therefore, for the therapeutic efficacy of MSC preparations and this warrants further investigation in future projects.

In agreement to our hypothesis we show that MSC properties are developmentally programmed in IUGR resulting in an enhanced capacity to differentiate into adipogenic and fibrogenic lineages at the expense of the osteogenic and chondrogenic lineages, as we observed in early passaged MSCs. This may underlie tissue development and inform on the development of therapies relevant to disease predisposition phenotypes observed during adulthood in IUGR individuals. Our results highlight important considerations when selecting MSC donors for regenerative medicine applications.

## Data Availability Statement

The raw data supporting the conclusions of this article will be made available by the authors, without undue reservation.

## Ethics Statement

Since the material used in the study was obtained post-mortem no licence is required following the guidelines of UK Home Office Animals 93 (Scientific Procedures) Act 1986.

## Author Contributions

EW, VA, YC-A, SD-J, and CS performed the experiments. EW, VA, and CE analyzed the data. CE and FD designed the project and wrote the manuscript. All authors contributed to the article and approved the submitted version.

## Conflict of Interest

The authors declare that the research was conducted in the absence of any commercial or financial relationships that could be construed as a potential conflict of interest.
