# Supplementary material for: Differentiation Potential of Mesenchymal Stem/Stromal Cells Is Altered by Intrauterine Growth Restriction
Source: Front Vet Sci. 2020 Nov 5;7:558905. doi: 10.3389/fvets.2020.558905 (PMC7676910; doi:10.3389/fvets.2020.558905)
Supplement: Supplementary file 1 [file Table_1.DOCX]

| Gene | Mean | | Standard Error of the Mean | | P value |
| --- | --- | --- | --- | --- | --- |
|  | IUGR-MSC | Normal-MSC | IUGR-MSC | Normal-MSC |  |
| **CD44** | 3.55 | 3.14 | 1.27 | 0.96 | 0.8 |
| **CD90** | 1.90 | 3.14 | 1.01 | 0.83 | 0.4 |
| **CD105** | 0.76 | 0.79 | 0.42 | 0.34 | 0.9 |

Supplementary Table 1. Gene expression values of CD44, CD90, CD105 for IUGR- and Normal-MSCs.
